# A Psychometric Evaluation of the Expanded Version of the Inventory of Depression and Anxiety Symptoms (IDAS-II) in Children and Adolescents

**DOI:** 10.1177/10731911231170841

**Published:** 2023-05-12

**Authors:** Matti Cervin, Carla Martí Valls, Stefan Möller, Andreas Frick, Johannes Björkstrand, David Watson

**Affiliations:** 1 Lund University, Sweden; 2 Uppsala University, Sweden; 3 University of Notre Dame, IN, USA

**Keywords:** IDAS-II, psychometrics, HiTOP, children, adolescents, internalizing, validation

## Abstract

The expanded version of the Inventory of Depression and Anxiety Symptoms (IDAS-II) is a self-report measure of 18 empirically derived internalizing symptom dimensions. The measure has shown good psychometric properties in adults but has never been evaluated in children and adolescents. A Swedish version of the IDAS-II was administered to 633 children and adolescents (*M*_age_ =16.6 [*SD* = 2.0]) and 203 adults (*M*_age_ = 35.4 [*SD* = 12.1]). The model/data fit of the 18-factor structure was excellent in both samples and measurement invariance across age groups was supported. All scales showed good to excellent internal consistency and psychometric properties replicated in the younger youth sample (< 16 years). Among youth, good convergent validity was established for all scales and divergent validity for most scales. The IDAS-II was better at identifying youth with current mental health problems than an internationally recommended scale of internalizing symptoms. In conclusion, the IDAS-II shows promise as a measure of internalizing symptoms in youth.

Mental health difficulties and mental disorders are major contributors to everyday impairment in children and adolescents (Gore et al., 2011). The most common and most impairing difficulties are internalizing symptoms, including fear, anxiety, and depression (Caspi et al., 2020; Polanczyk et al., 2015). A vast majority of all individuals who develop mental disorders develop their first disorder before adulthood (Caspi et al., 2020) and the peak age of onset is 14 years (Solmi et al., 2022). The early development of mental health problems makes research with children and adolescents important, and adequate measurement of symptoms is key to improving our understanding of the development and impact of mental disorders among youth.

Mental disorders in youth are commonly classified according to the *Diagnostic and Statistical Manual of Mental Disorders* (*DSM*; American Psychiatric Association, 2022), where subsets of symptoms are used as criteria for different disorders (e.g., major depression, post-traumatic stress disorder [PTSD], social anxiety disorder). Based on symptom presentation, distress, and functional impairment, an individual is considered to either meet or not meet criteria for a specific disorder. Albeit a useful framework within health services, where treatment decisions are often categorical and where diagnoses are used as treatment indicators and prognostic markers, there are downsides of the current diagnostic classification system. First, discrete disorder categories do not align with strong evidence indicating that mental health difficulties are best viewed as dimensional continua without clear thresholds between normal and abnormal (Kotov et al., 2017). Second, psychiatric comorbidity is ubiquitous in the mental health field, that is, a majority of all individuals who meet criteria for one psychiatric disorder also meet criteria for another disorder, challenging the notion of discrete disorders (Caspi et al., 2014). Third, there is substantial heterogeneity within disorders and overlapping criteria between disorders (Newson et al., 2021). These challenges, along with evidence of questionable reliability, discriminant validity, and content validity for psychiatric diagnoses have led to the development of alternative models aimed to explain the structure of mental health symptoms (Insel et al., 2010; Watson et al., 2022).

One alternative (or complementary) model is the Hierarchical Taxonomy of Psychopathology (HiTOP) (Kotov et al., 2017), which attempts to overcome the above challenges by addressing mental health difficulties through a quantitative and hierarchical approach. The HiTOP model is hierarchically based because although virtually all forms of psychopathology are positively correlated with one another (as demonstrated by the existence of an overarching *p* factor), some forms are more strongly related than others. For instance, social anxiety is more strongly related to depressed mood than it is to antisocial behavior. In a hierarchical model, more strongly correlated variables are placed closer together (e.g., social anxiety and depressed mood both are indicators of the internalizing dimension, whereas antisocial behavior is placed within the externalizing dimension).

In HiTOP, psychopathological phenomena are classified into different levels of specificity that move from specific signs, symptoms, and behaviors (e.g., insomnia or irritability), to tightknit symptom components (e.g., agoraphobia, social anxiety), dimensional syndromes (e.g., distress, fear), broad spectra (e.g., internalizing, externalizing, thought disorder), and finally a general psychopathological factor, often referred to as the *p* factor, which indicates a broad propensity toward many different types of mental health difficulties (Caspi et al., 2014; Watson et al., 2022). In youth with anxiety disorders, the *p* factor has been shown to be a stronger predictor of long-term outcomes than *DSM* diagnoses and other established risk factors, such as poor family functioning (Cervin, Norris, et al., 2020), lending support for the possible benefits of addressing youth mental health symptoms using an hierarchical dimensional framework as suggested by HiTOP.

In the HiTOP system, symptoms of depression and anxiety are included under the broad internalizing spectrum, which also includes obsessive–compulsive, trauma and stressor-related, personality disorder, and eating disorder symptoms (Watson et al., 2022). To assess symptoms within this spectrum, Watson et al. (2007) developed the Inventory of Depression and Anxiety Symptoms (IDAS), a 64-item self-report measure assessing various symptoms related to the dimensional syndromes of *fear* and *distress*. The original IDAS had 11 non-overlapping scales: dysphoria, insomnia, lassitude (or fatigue), suicidality, appetite loss, appetite gain, ill temper (i.e., anger or hostility), well-being, panic, social anxiety, and traumatic intrusions (Watson et al., 2007). A goal of the measure was to provide a broad screening tool for internalizing disorders. Broad screening is important because of the vast comorbidity between internalizing symptoms and disorders. In addition, a single measure would eliminate the methodological problems stemming from assessing different symptoms using different scales (Irak & Albayrak, 2020).

The IDAS-II (Watson et al., 2012) expanded upon the original IDAS with the goal of including additional symptoms of anxiety (claustrophobia), PTSD (traumatic avoidance), obsessive–compulsive disorder (OCD) (cleaning, ordering, and checking), and bipolar disorder (BD) (euphoria and mania). The IDAS-II encompasses 99 items that yield 18 non-overlapping scales.^1^ Interestingly, many of the symptom scales of the IDAS-II (e.g., mania, checking, ordering, cleaning) may relate to both the internalizing and thought disorder spectra of HiTOP. According to Watson et al. (2012), the resulting overarching factor structure of IDAS-II includes three components: (a) *Distress*, including symptoms of major depressive disorder (MDD), generalized anxiety disorder (GAD), and PTSD; (b) *Obsessions/Fear*, grouping symptoms of claustrophobia, social anxiety, and OCD; and (c) *Positive Mood*, defined by well-being and euphoria.

Several translations of the IDAS-II exist and the measure is extensively used in research, including in research with children and adolescents (Bartlett et al., 2019; B. D. Nelson et al., 2015), but no psychometric evaluation of the scale has been conducted with youth. Adult evaluations support that the proposed 18-dimension model of the measure fits self-reported data well, that is, self-reported symptoms within the proposed dimensions co-occur in a way that is predicted by the model (De la Rosa-Cáceres et al., 2020; Irak & Albayrak, 2020; Weitzner et al., 2020; Wester et al., 2022). The IDAS-II scales have shown good internal consistency (most estimates above a Cronbach’s alpha of .80) (De la Rosa-Cáceres et al., 2020; Weitzner et al., 2020) and temporal stability (with most test–retest correlations being above .70) (De la Rosa-Cáceres et al., 2020; Irak & Albayrak, 2020; Watson et al., 2012; Weitzner et al., 2020). Evaluations have also supported that IDAS-II shows adequate convergent and discriminant validity when compared with other self-report and interview-based measures of internalizing symptoms (De la Rosa-Cáceres et al., 2020; Stasik-O’Brien et al., 2019; Watson et al., 2012; Weitzner et al., 2020). In terms of criterion validity, the IDAS-II subscales present generally high correlations (*r*s ∼.50) with interview-based measures disorders included in the *Diagnostic and Statistical Manual of Mental Disorders* (4th ed.; *DSM-IV*; American Psychiatric Association, 1994) (Watson et al., 2012). Prior studies have also reported adequate discrimination between clinical and non-clinical samples, indicating medium to large effect sizes for most scales (Cohen’s *d*s > .70), but smaller effects for the OCD scales, which has been interpreted as reflecting common subclinical manifestations of OCD-related symptoms and behaviors in the general population (Irak & Albayrak, 2020; Watson et al., 2012). Furthermore, scores on the euphoria scale have been found to be higher in non-clinical than in clinical samples, raising some concerns about the complex relationship between euphoria and BD (Watson et al., 2012). Finally, an overarching three-factor structure (distress, obsessions/fear, and positive mood) has largely been supported (De la Rosa-Cáceres et al., 2020; Irak & Albayrak, 2020; Weitzner et al., 2020; Wester et al., 2022).

In sum, previous research indicates that IDAS-II is a useful assessment tool in both research and clinical settings, and it has unique characteristics given that it assesses a broad range of empirically derived symptom dimensions using a single measure. The scale is based on the HiTOP framework and can support research using this taxonomy. Nevertheless, its psychometric properties in children and adolescents are unknown. Prior research into the hierarchical dimensional structure of psychopathology has largely supported similar dimensions and hierarchical structures across age groups (Hankin et al., 2017; Martel et al., 2017; Patalay et al., 2015), which would suggest that IDAS-II may be valid also for children and adolescents, but empirical evaluations are needed. Because mental health problems typically begin during childhood or adolescence, theoretically informed measures that can be used in this age group are important for both clinical and research purposes. The aim of this study is to conduct a comprehensive psychometric evaluation of a Swedish version of the IDAS-II in children and adolescents. In line with adult findings, we expect the scale to show adequate psychometric properties.

## Method

### Participants and Procedure

Child and adolescent participants were recruited through social media and school platforms and completed an anonymous online survey that included the IDAS-II and other measures of mental health. Participants provided informed assent before completing the online survey. The survey was hosted by Sunet Survey, which is an online questionnaire research tool available through Lund University. Only basic sociodemographic information was collected to secure anonymity. The only inclusion criterion was being between 10 and 19 years of age. No exclusion criteria were applied. The youth sample was collected in two waves. The first data collection (Survey 1) was conducted between September and December 2018. The second data collection (Survey 2) was conducted between January and May 2022. The youth study was approved by the regional ethics committee as part of an amendment to a larger clinical research project (Dnr: 2018/668). Based on the strong psychometric properties of the IDAS-II in adult evaluations and since we expected that the measure would work also with youth, we set out to recruit five youth participants per item, yielding a target sample size of 495 participants. Two surveys were needed to reach this number.

The adult sample was recruited using social media advertisements and was part of a study examining emotional processing and psychopathology. Participants were required to be aged 18 years or older and have self-reported normal or corrected-to-normal vision and hearing. However, to produce samples that did not overlap in age, we eliminated participants from this sample who were 18 or 19 years old (*n* = 9). Participants provided informed consent prior to participation and were reimbursed with a gift card worth 200 SEK (approximately 20 USD). Consent and IDAS-II data were collected online using REDCap (Research Electronic Data Capture), a secure, web-based software platform designed to support data capture for research studies (Harris et al., 2019). The study was approved by the Swedish Ethical Review Authority (2020-05777). The adult sample was a convenience sample. Table 1 presents sociodemographic characteristics of the youth and adult samples.

**Table 1:** Sample Characteristics.

Youth sample			Adult sample		
*n*		633	*n*		203
Age		Age			
	*M* (*SD*)	16.56 (1.98)		*M* (*SD*)	35.39 (12.09)
	Min.–max.	10–19		Min.–max.	20–71
	10 to <12 years, *n* (%)	29 (4.6%)		20 to <30 years, *n* (%)	83 (39.2%)
	12 to <14 years, *n* (%)	22 (3.5%)		30 to <40 years, *n* (%)	53 (25.0%)
	14 to <16 years, *n* (%)	68 (10.7%)		40 to <50 years, *n* (%)	37 (17.5%)
	16 to <18 years, *n* (%)	282 (44.5%)		50+ years, *n* (%)	30 (14.2%)
	18 to <19 years, *n* (%)	232 (36.7%)			
Gender			Gender		
	Girl, *n* (%)	433 (68.4%)		Female, *n* (%)	170 (87.2%)
	Boy, *n* (%)	182 (28.8%)		Male, *n* (%)	25 (12.8%)
	Other, *n* (%)	6 (0.9%)	*Highest education*		
	Unsure, *n* (%)	10 (1.6%)		University, *n* (%)	95 (54.9%)
	Do not want to report, *n* (%)	2 (0.3%)		Short post high school, *n* (%)	11 (6.4%)
Current mental health problems				Theoretical high school, *n* (%)	27 (15.6%)
	Yes, *n* (%)	321 (50.7%)		Practical high school, *n* (%)	33 (19.1%)
	No, *n* (%)	312 (49.3%)		No high school, *n* (%)	7 (4.0%)
Current treatment for mental health problems					
	Yes, *n* (%)	119 (18.8%)			
	No, *n* (%)	514 (81.2%)			
Previous mental health problems					
	Yes, *n* (%)	400 (63.2%)			
	No, *n* (%)	233 (36.8%)			
Previous treatment for mental health problems					
	Yes, *n* (%)	217 (34.3%)			
	No, *n* (%)	416 (65.7%)			

### Measures

#### Expanded Version of the Inventory of Depression and Anxiety Symptoms

The IDAS-II includes 99 items and each item is scored on a 5-point Likert-type scale (1 = Not at all to 5 = Extremely) (Watson et al., 2012). The measure yields 18 subscales (dysphoria, lassitude, insomnia, suicidality, appetite loss, appetite gain, well-being, ill temper, mania, euphoria, panic, social anxiety, claustrophobia, traumatic intrusions, traumatic avoidance, checking, ordering, and cleaning). As described in the introduction, the measure has shown adequate psychometric properties in adults (G. H. Nelson et al., 2018; Watson et al., 2012) but has never been evaluated using youth samples. The IDAS-II was translated to Swedish by a team of mental health experts, including the first author (MC) of this study. Translated items were presented to children and adolescents who provided feedback on clarity and understandability. This led to clarifications of certain words and phrases (e.g., self-conscious, accomplished, lose temper), which were added within parentheses. No other age adjustments/adaptations were made. The Swedish translation was then back translated into English by a bilingual clinical psychologist not involved in the first translation. The back translated version was reviewed by the original scale developer (Prof. David Watson) and some items were discussed and corrected before the final version of the scale was approved. The youth and adult samples completed the same Swedish version of the IDAS-II.

#### Revised Child Anxiety and Depression Scale

The Revised Child Anxiety and Depression Scale (RCADS) includes 47 items (Chorpita et al., 2000). Each item is scored using a 0 to 3 scale with higher scores indicating more frequent symptoms. The RCADS yields six subscales: depression, social anxiety, panic, generalized anxiety, separation anxiety, and OCD. The measure has shown adequate psychometric properties (e.g., internal consistency in the form of Cronbach’s *a* > .70 and adequate model/data for the proposed six-factor model) in several studies (Piqueras et al., 2017), including in Swedish youth (Cervin, Veas, et al., 2022), and is an internationally recommended measure for the assessment of internalizing symptoms in children and adolescents (Krause et al., 2021). In the present study, the internal consistency (Cronbach’s alpha, *a*) of each RCADS scale was adequate: depression (*a* = .92), social anxiety (*a* = .92), panic (*a* = .94), generalized anxiety (*a* = .87), separation anxiety (*a* = .87), and OCD (*a* = .88). We used the RCADS to examine convergent and divergent validity of the IDAS-II scales. The RCADS was only administered in the Youth Survey 1.

#### Child Sheehan Disability Scale

Functional impairment stemming from mental health difficulties was assessed using the Child Sheehan Disability Scale (CSDS), which includes impairment in three areas: school functioning, family functioning, and social functioning (Whiteside, 2009). Each area is rated using a 0 to 10 scale with higher scores indicating greater impairment. Prior research has shown that CSDS has adequate psychometric properties (e.g., internal consistency > .70), strong convergent and discriminant validity (i.e., moderate to strong correlations with related constructs), and known groups validity (i.e., ability to discriminate youth with and without mental disorders) (Whiteside, 2009), including in Swedish settings (Soler et al., 2021). The three areas are usually pooled into an overall impairment score and were so also in the present study; the Cronbach’s alpha was adequate (*a* = .89). The CSDS was administered in Youth Surveys 1 and 2 but not in the adult sample.

### Statistical Analysis

All statistical analyses were conducted in R Studio (version 2021.09.0) (R Core Team, 2020). First, we used confirmatory factor analysis (CFA) to test the proposed factor structure of the IDAS-II that includes 18 first-order factors that are allowed to correlate freely. The CFAs were conducted using the R library *lavaan* (Rosseel, 2012). Because the IDAS-II items are ordinal, we used the diagonally weighted least squares estimator that can model non-normal responses as indicators of a normally distributed latent factor. Model/data fit was evaluated by inspecting four fit indices: comparative fit index (CFI), Tucker–Lewis index (TLI), root mean square error of approximation (RMSEA), and standardized mean square residual (SRMR). An RMSEA below 0.06, an SRMR below 0.08, and CFI and TLI estimates greater than 0.90 are indicative of acceptable model–data fit; CFI and TLI estimates above 0.95 are indicative of good model–data fit (Hu & Bentler, 1999). We estimated scaled fit indices because of the ordinal nature of the data. The proportion of missing data was low (Youth Survey 1, 2.9%; Youth Survey 2, 2.6%; adults, 0.0%—the digital system used with the adult sample did not allow for skipping items) and missing data were handled using pairwise deletion. Finally, because the youth sample was predominantly in the older adolescent age range, we split the youth sample into those under 16 years of age (*n* = 119) and those 16–19 years of age (*n* = 514), fitted the model separately in each sample, and examined model/data fit.

To examine whether the proposed 18-factor IDAS-II structure showed measurement invariance across youth and adults, we conducted multi-group confirmatory factor analysis (MG-CFA) using *lavaan*. MG-CFA can empirically test whether a measure psychometrically assesses the same constructs across different groups (e.g., children/adults, different countries), which is a prerequisite to compare factor scores across these groups. In a first step, we used MG-CFA to test for configural invariance. Configural invariance indicates that the same items load onto the same factors across groups. We then tested for scalar invariance, which indicates the same thresholds and factor loadings across groups. The common step of metric invariance was omitted because when items are ordinal, loadings and intercepts jointly define item function and thus should be freed/restrained in tandem (Sass et al., 2014). Finally, we tested for strict invariance, which indicates the same factor loadings, thresholds, and residuals across groups. MG-CFA is conducted by restraining model parameters in a stepwise fashion according to the three steps described above and invariance is evaluated by inspecting change in fit indices. We interpreted a drop in CFI (ΔCFI) <.01 as an indicator of invariance (Kim et al., 2017). In all MG-CFA analyses, two items were omitted because they had zero observations in some categories in the adult sample (Item 22, “I cut or burned myself on purpose” and Item 90, “I was afraid of tunnels”).

Internal consistency (i.e., a quantification of the amount of random measurement error) for the items of each scale was examined using Cronbach’s alpha and McDonald’s omega. Internal consistency coefficients above 0.70 are often considered adequate but coefficients above 0.80 are preferable. McDonald’s omega was calculated as it is less restrictive in its assumptions compared with the more widely used Cronbach’s alpha (Dunn et al., 2014). Coefficients above .90 were considered excellent, coefficients between .80 and .90 were considered good, coefficients between .70 and .80 adequate, and coefficients below .70 problematic.

CFA does not include systematic examination of overarching factor structures, that is, how the first-order factors of the IDAS-II relate to each other. Instead, to explore overarching factor structures, we conducted exploratory factor analysis (EFA) using the 18 IDAS-II scales as input. The Kaiser–Meyer–Olkin (KMO) test values were used to examine whether the full set of scales as well as each individual scale was suitable for EFA. KMO values indicate the proportion of variance in variables that might be explained by latent factors and values above .80 are considered to indicate that EFA is well suited. We used parallel analysis implemented in the R library *psych* to determine the number of factors to retain during EFA. Factors were extracted using principal axis factoring and promax rotation. Principal axis factoring was used because it does not include a multivariate normal assumption and promax rotation was used because the IDAS factors are known to be significantly correlated with one another.

To examine convergent and divergent validity in the youth sample, we estimated associations (correlations) between the IDAS-II and RCADS scales. The IDAS-II and RCADS both include scales pertaining to depressive symptoms (IDAS-II: dysphoria; RCADS: depression), social anxiety, panic, and OCD (IDAS-II: checking, ordering, cleaning; RCADS: OCD). To test whether the correlation between scales measuring similar constructs (e.g., IDAS-II dysphoria and RCADS depression) was stronger than between scales of dissimilar constructs (e.g., IDAS-II dysphoria and RCADS OCD), we used the method presented in the work of Hittner et al. (2003), which is implemented in the R library *cocor*. We compared correlations using the following convergent associations: IDAS-II dysphoria with RCADS depression; IDAS-II social anxiety with RCADS social anxiety; IDAS-II panic with RCADS panic; and IDAS-II checking, ordering, and cleaning with RCADS OCD. Correlations above .50 were considered to indicate adequate convergent validity.

Associations between the IDAS-II scales and functional impairment in the youth sample were examined by correlating each of the IDAS-II scales with the CSDS. Correlations of ∼.10 were considered small, ∼.30 medium, and >.50 large. The ability of the IDAS-II scales to discriminate between those who self-identified as suffering from current mental health problems from those who did not experience such difficulties was examined by conducting independent samples t-tests and quantifying differences using Cohen’s *d*, where *d*s between .50 and .80 were considered to indicate moderate differences and *d*s > .80 to indicate large differences.

Finally, we again used the self-report item in which youth participants reported on whether they experienced current mental health problems and examined the degree to which the IDAS-II and RCADS could be used to correctly classify participants who affirmed this item. This was considered a test of the screening properties of each measure. Survey 1 was used in these analyses since the RCADS was not included in Survey 2. We randomly split the sample 70/30% and estimated regression coefficients for all symptom scales using a logistic regression model based on the subsample that included 70% of the participants. The item indicating current mental health symptoms was used as the dependent variable. We then classified participants (current mental health problems or not) in the remaining 30% of the sample using these coefficients and examined sensitivity (proportion of participants reporting mental health symptoms who were classified as having mental health symptoms), specificity (proportion of participants reporting no mental health symptoms who were classified as having no mental health symptoms), the positive predictive value (PPV; proportion of participants classified as having mental health symptoms who reported mental health symptoms), and the negative predictive value (NPV; proportion of participants classified as not having mental health symptoms who reported not having mental health symptoms) of the IDAS-II and RCADS. No formal statistical comparison of classification performance was conducted because of low power; rather, we compared the point estimates.

Statistical code and the youth data used in the present study are freely available on the Open Science Framework repository (https://osf.io/n5um8/). The adult data are not freely shared because of regulations at one of the participating universities but it is available upon reasonable request. The study was not preregistered, but all factor analyses were based on the model proposed in the original validation of the IDAS-II (Watson et al., 2012). Questions about analyses and/or data will be answered by the corresponding author.

## Results

### Data Quality

All data were screened for straightlining responses (i.e., when a respondent gives identical or nearly identical responses to all items) by estimating the individual variation around the individual means for scales as well as the full measure, which was then manually screened. No cases of straightlining were identified.

### Model/Data Fit and Internal Consistency

Model/data fit in the youth sample (both in the full sample, and in the younger and older age groups) and the adult sample as well as for Surveys 1 and 2 for the youth sample are presented in Table 2. Model/data fit was good according to all fit indices in all samples and surveys (CFI/TLI = .94–.96, RMSEA = .03–.04, SRMR = .06–.09). Strict invariance between youth and adults was established (i.e., the drop in CFI did exceed .01 with increasing constraints; see bottom of Table 2). Standardized factor loadings in the youth and adult samples are presented in the Supplemental Table S1.

**Table 2:** Model/Data Fit for the Original IDAS-II Factor Structure Using the Different Samples and Measurement Invariance Tests for Youth Versus Adults.

Sample	χ2	*df*	*p*	CFI	TLI	RMSEA	SRMR
Full youth sample (*n* = 633)	8,354	4,599	<.001	0.94	0.94	0.04	0.06
Youth sample, Survey 1 (*n* = 408)	7,042	4,599	<.001	0.95	0.94	0.04	0.07
Youth sample, Survey 2 (*n* = 225)	5,478	4,599	<.001	0.95	0.95	0.03	0.08
Adult sample (*n* = 202)	5,431	4,599	<.001	0.95	0.95	0.03	0.08
Younger youth sample (*n* = 119)	5,091	4,599	<.001	0.96	0.96	0.03	0.09
Older youth sample (*n* = 514)	7,657	4,599	<.001	0.94	0.94	0.04	0.07
Invariance tests (youth vs. adults)							
Configural	12,581	8,812	<.001	0.95	0.95	0.03	0.07
Scalar	12,510	9,085	<.001	0.95	0.95	0.03	0.07
Strict	12,626	9,182	<.001	0.96	0.95	0.03	0.07

*Note.* CFI = comparative fit index; TLI = Tucker–Lewis index; RMSEA = root mean square error of approximation; SRMR = standardized mean square residual.

The internal consistency of the items of each scale in each sample is presented in Supplemental Table S2 (full youth and adult samples plus Surveys 1 and 2 for the youth sample) and Supplemental Table S3 (younger and older youth samples). Internal consistency was good to excellent (alpha/omega > .80 and > .90, respectively) for all scales in all samples except for appetite gain, euphoria, and checking which were adequate (alpha/omega > .70 to <.80) in some samples and good in others (alpha/omega >.80 to <.90). Very similar internal consistencies were found across youth and adults as well as separately for Surveys 1 and 2 and for the younger and older age groups within the youth sample.

#### Overarching Factor Structure

We estimated the correlation matrix for the 18 IDAS-II scales in youths and adults, respectively. The overall KMO test value for youths was 0.92 with the lowest dimension-level value being 0.55 (euphoria) and the second lowest being 0.86 (well-being and ordering). For adults, the overall KMO test value was 0.88, with the lowest value being 0.56 (euphoria) and the second lowest being 0.71 (appetite gain). Thus, overall, the IDAS-II scales were well suited for EFA.

We examined the number of factors to extract. For both youth and adult data, parallel analysis suggested five factors. We compared three-, four-, five-, six-, and seven-factor solutions in both age groups. To find the most suitable solution, we examined three different properties: (a) cross loadings, (b) correlations among dimensions included in each factor, and (c) total variance explained. A four-factor solution received most support in youth and a three-factor solution in adults. The four-factor solution in youth explained 54.0% of the covariance among the 18 dimensions. In adults, the three-factor solution explained 53.2% of the covariance.

**Figure 1.**
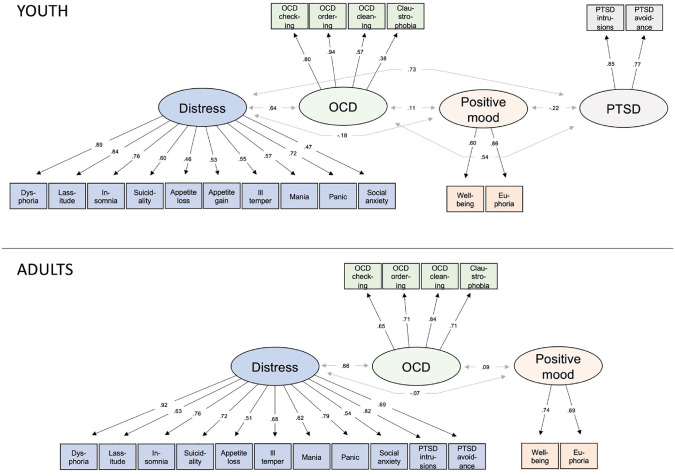
Factor Structures for Youth and Adults With Factor Loadings and Covariance Among Overarching Dimensions. *Note.* OCD = obsessive–compulsive disorder; PTSD = post-traumatic stress disorder.

The respective factor solutions are presented in Figure 1 together with factor loadings and covariances among the factors. The overarching factor structures were identical with two exceptions. In adults, the two PTSD dimensions (intrusions and avoidance) loaded onto the distress factor while in youth they formed their own factor. Furthermore, appetite gain did not have a factor loading > .30 on any factor in adults, whereas it loaded onto the distress factor in youth. In youth, only well-being had a cross loading > .30 (a negative loading on the distress factor). In adults, well-being (negative loading on the distress factor), mania (positive loading on the positive mood factor), and social anxiety (positive loading on the OCD factor) had cross loadings > .30.^2^

#### Convergent and Divergent Validity

Convergent and divergent validity of the IDAS-II scales in the youth sample, assessed using correlations between the IDAS-II scale and RCADS scales, are presented in Table 3. With regard to convergent validity, each of the IDAS-II scales correlated moderately to strongly with the corresponding RCADS scale. Further, for convergent validity, the IDAS-II dysphoria scale showed a strong correlation with the RCADS depression scale, and this correlation was statistically significantly stronger than the correlation between the IDAS-II dysphoria scale and each of the other RCADS scales. Similar results were found for the IDAS-II checking and ordering scales, which showed significantly stronger correlations with the RCADS OCD scale than with all other RCADS scales. The IDAS-II cleaning scale also showed a significantly stronger correlation with the RCADS OCD scale than with all other RCADS scales except social anxiety. The IDAS-II panic scale was significantly more strongly correlated with the RCADS panic scale than with all other RCADS scales except for the RCADS depression scale. The IDAS-II social anxiety scale was significantly more strongly correlated with the RCADS social anxiety scale than with the RCADS OCD and separation anxiety scales, but no significant differences were found compared with the correlations with the other RCADS scales. Finally, we estimated correlations between the IDAS-II well-being scale and the RCADS scales. The well-being scale correlated negatively with all RCADS scales with the strongest negative correlation emerging in relation to RCADS depression.

**Table 3:** Convergent and Divergent Correlations Between IDAS-II and RCADS. The Corresponding Correlations are Highlighted in Bold.

Scale	RCADS depression	RCADS social anxiety	RCADS panic	RCADS OCD	RCADS GAD	RCADS separation anxiety
IDAS-II dysphoria	**0.69**	0.57^a^	0.52^a^	0.48^a^	0.58^a^	0.41^a^
IDAS-II social anxiety	0.51^c^	**0.48**	0.42^c^	0.36^a^	0.46^c^	0.30^a^
IDAS-II panic	0.58^b^	0.46^a^	**0.54**	0.43^a^	0.47^a^	0.34^a^
IDAS-II checking	0.35^a^	0.33^a^	0.32^a^	**0.42**	0.36^a^	0.24^a^
IDAS-II ordering	0.28^a^	0.26^a^	0.25^a^	**0.35**	0.29^a^	0.19^a^
IDAS-II cleaning	0.31^a^	0.32^c^	0.28^a^	**0.37**	0.29^a^	0.22^a^
IDAS-II well-being	−0.54	−0.39	−0.34	−0.30	−0.38	−0.26
Broader dimensions						
IDAS-II distress	0.71	0.56	0.57	0.50	0.58	0.40
IDAS-II OCD	0.48^c^	0.44^a^	0.43^a^	**0.50**	0.46 ^c^	0.31^a^
IDAS-II PTSD	0.55	0.47	0.49	0.46	0.48	0.31
IDAS-II positive mood	−0.04	0.01	0.03	0.07	0.00	0.03

*Note.* RCADS = Revised Child Anxiety and Depression Scale; OCD = obsessive–compulsive disorder; GAD = generalized anxiety disorder; IDAS-II = expanded version of the Inventory of Depression and Anxiety Symptoms; PTSD = post-traumatic stress disorder.

a The correlation is statistically significantly smaller in the expected direction. ^b^ A statistically significantly stronger correlation in the unexpected direction. ^c^ No statistically significant differences for the correlation coefficients.

We also examined the convergent/divergent validity of the four broader IDAS-II dimensions in youth (distress, OCD, PTSD, and positive mood) by correlating predicted factor scores with the RCADS scales. Results are at the bottom of Table 3. The broad distress dimension was moderately to strongly associated with all RCADS scales and the broad PTSD dimension was moderately associated with all RCADS scales. For the broad OCD dimension, we expected that it would be most strongly associated with the broad OCD RCADS scale, which was confirmed compared with RCADS panic, RCADS social anxiety, and RCADS separation anxiety. The broad positive mood dimension had small and negligible correlations with all RCADS scales.

See Table 4 for correlations between the IDAS-II scales and self-reported functional impairment in the youth sample. All scales correlated statistically significantly with functional impairment except euphoria and the significant correlations were in the moderate to strong range. Similarly, all scales discriminated, in a statistically significant way, those who self-identified as having current mental health problems from those without such problems, with large between-group differences for all scales except euphoria (where those without mental health symptoms had higher scores), claustrophobia, and the three OCD scales, where differences were small to moderate in magnitude.

**Table 4:** Association Between Each IDAS-II Scale and Functional Impairment and the Difference Between Those Self-Identifying as Having Current Mental Health Problems Versus Those Without Such Problems Expressed as Cohen’s *d*s.

Scale	Correlation with functional impairment	Difference for current mental health problems vs. no current problems Cohen’s *d*
Dysphoria	.75**	1.70**
Panic	.59**	1.18**
Suicidality	.57**	1.06**
Lassitude	.56**	0.96**
Ill temper	.52**	1.03**
Social anxiety	.52**	1.00**
Traumatic avoidance	.52**	0.91**
Appetite loss	.51**	0.94**
Traumatic intrusions	.51**	1.01**
Insomnia	.50**	0.97**
Mania	.46**	0.84**
Claustrophobia	.38**	0.66**
Checking	.34**	0.59**
Appetite gain	.27**	1.07**
Ordering	.26**	0.37**
Cleaning	.26**	0.41**
Euphoria	−.07	−0.23*br25-1073191
Well-being	−.55**	−1.19**

*Note.* Scales are Ordered According to the Strength of Their Association With Functional Impairment. IDAS-II = expanded version of the Inventory of Depression and Anxiety Symptoms.

** *p* < .001. *p < .01.

### Classification Performance

The 18 IDAS-II scales correctly classified 81.5% of all youth who self-identified as having ongoing mental health symptoms (i.e., a sensitivity of 81.5%). Furthermore, among those who were classified as having ongoing mental health symptoms, 81.5% were correctly classified (a PPV of 81.5%). The specificity was 78.2% and the NPV was also 78.2%. In contrast, the six RCADS scales had a sensitivity of 78.8%, a PPV of 74.3%, a specificity of 67.9%, and an NPV of 73.1%. In Supplemental Table S4, we present the 2 by 2 tables that were used to estimate the above reported classification performance.

## Discussion

The aim of this study was to examine the psychometric properties of the IDAS-II in children and adolescents. Based on evidence suggesting that the structure of psychopathological symptoms is similar in youths and adults, we expected that the measure would show good psychometric properties in youths. The findings of the present study confirmed our expectations.

First, the model/data fit of the proposed factor structure with 18 first-order dimensions was good to excellent according to all fit indices. The fit indices were also very similar in the youth and adult samples, and in the younger and older youth samples. These findings indicate that the items of the IDAS-II, which were developed for adults, group together in a very similar way in children and adolescents. Second, when comparing the factor loadings across the youth and adult samples, remarkable similarities were found and strict invariance was established, establishing that the IDAS-II behaves very similarly in youth as compared with adult samples. Third, the internal consistency of the items of each scale was adequate to excellent and again very similar results emerged for youths and adults.

Although adequate psychometric properties of the IDAS-II in youths were expected, the almost completely preserved psychometric properties, compared with estimates from adult samples, and excellent properties also in the youngest age group, were somewhat surprising. Several items of the IDAS reflect complex internal experiences and no items were changed in the youth version, although clarifications of some terms were added. Children and adolescents are expected to have more difficulties than adults in operationalizing and reporting internal experiences, which potentially could affect the psychometric properties of the measure. We found no evidence of such effects in the present study and it may be that the careful and step-wise development of IDAS-II (Watson et al., 2012) has resulted in final pools of items that are easy to understand and clearly load onto their expected factors. With regard to variability in internal consistency, our results were similar to those from previous evaluations, showing the lowest internal consistency for appetite gain (Irak & Albayrak, 2020) and euphoria (Watson et al., 2012; Wester et al., 2022). However, the scale with the lowest internal consistency in the youth sample in this study (appetite gain) still had adequate internal consistency and all other scales showed good to excellent internal consistency.

We also explored the overarching factor structure defined by the 18 symptom dimensions of the IDAS-II and similar results again emerged for youths and adults, which were also largely in line with previous adult evaluations (De la Rosa-Cáceres et al., 2020; Irak & Albayrak, 2020; Watson et al., 2012; Weitzner et al., 2020; Wester et al., 2022). Three highly similar factors emerged in both youth and adults (distress, positive mood, and OCD), and a fourth for youth (PTSD). Few cross loadings were present and all but two scales in the youth sample and all scales in the adult sample loaded > .50 onto its proposed factor. Taken together, several studies have now suggested that the IDAS-II dimensions can be sorted according to overarching symptom dimensions and this study suggests that these overarching dimensions are broadly similar in youths and adults. However, future research is needed to test whether the proposed overarching structure shows adequate model/data fit when tested in a confirmatory fashion.

After establishing sound psychometric properties of the IDAS-II in youths and examining its overarching structure, we examined convergent and divergent validity by correlating both the first-order scales and the overarching dimensions with the subscales from the RCADS. The RCADS is an internationally recommended measure of internalizing symptom dimensions in youth (Krause et al., 2021) and we evaluated whether scales assessing similar symptoms (i.e., corresponding scales) were at least moderately correlated and more strongly correlated than scales assessing non-overlapping symptoms (i.e., non-corresponding scales). Corresponding scales were used to examine convergent validity. These included the dysphoria scale of IDAS-II—a scale assessing core emotional and cognitive symptoms of depression and anxiety (Watson et al., 2012)—and the depression scale of RCADS; the social anxiety scales of both measures; the panic scales of both measures; and the OCD scales of both measures. Findings indicated strong correlations for all corresponding scales except between the OCD scales of the two measures, which showed moderate correlations. Lower correlations between the OCD scales of IDAS-II and the OCD scale of RCADS are expected since OCD symptoms are heterogeneous in youth, both topographically and functionally (Cervin, Miguel, et al., 2022; Cervin, Perrin, Olsson, Aspvall, et al., 2020; Cervin, Perrin, Olsson, Claesdotter-Knutsson, & Lindvall, 2020). This heterogeneity is appreciated by the IDAS-II, which assesses checking, ordering, and cleaning, dimensions which largely corresponds to the three major symptom dimensions of OCD in youth, namely, disturbing thoughts and checking, symmetry and ordering, and contamination and washing (Cervin, Miguel, et al., 2022). The RCADS, on the other hand, only includes a single OCD scale that pools together OCD symptoms of different types.

Of the 30 indicators, 25 supported divergent validity for the IDAS-II scales, that is, that the corresponding scales of the IDAS-II and RCADS correlated significantly more strongly than non-corresponding scales. The scale with the weakest evidence for divergent validity was social anxiety. The reasons for this are unclear, and it is important to point out that it may reflect problems in this IDAS-II scale and/or one or more RCADS scales. Future research should revisit this issue, which will be best examined by including diagnostic interviews and more in-depth information about the nature of psychopathological problems in participants. Finally, the well-being scale of IDAS-II showed moderate to strong negative correlations with all RCADS scales, indicating that this scale is associated with the absence or reduced frequency of a broad range of internalizing mental health symptoms in youth.

We also examined correlations between the overarching IDAS-II dimensions derived from exploratory factor analysis and the RCADS scales. The overarching distress, OCD, and PTSD dimensions all correlated moderately to strongly with all RCADS scales, with the distress dimension showing the strongest correlations. As expected, the correlation between the overarching OCD dimension and the RCADS OCD scale was stronger than the correlations between the three separate IDAS-II OCD scales and the RCADS OCD scale. The overarching positive mood dimension was very weakly correlated with the RCADS scales, making it uncertain what this dimension reflects. The IDAS-II well-being scale showed clear negative correlations with all RCADS scales and, although well-being and euphoria grouped together into an overarching factor using exploratory factor analysis, this combination yielded unclear relations to convergent validators. Of note, during EFA, the euphoria scale stood out as the scale showing least amount of shared variance with other scales.

Further support for construct validity was found when we correlated the IDAS-II scales with self-reported mental health-related functional impairment. Findings showed that all scales except euphoria correlated moderately to strongly with functional impairment. All scales also discriminated those who self-identified as having current mental health difficulties from those who self-identified as not having such difficulties. These results indicate that although the IDAS-II includes a long list of symptom dimensions, virtually all these dimensions are related to functional impairment in youth, which is important both for the construct validity of the measure and for its clinical usefulness.

Having said that, the euphoria scale was not significantly correlated with impairment and only yielded a small between-group difference with *higher* scores among those without current mental health problems. Although the euphoria scale was developed to assess a symptom dimension of BD, it has previously showed weak convergent validity (De la Rosa-Cáceres et al., 2020; Weitzner et al., 2020) and in the present study it discriminated significantly between those with and without mental health difficulties but in the opposite direction of what was expected (i.e., higher scores among those without mental health difficulties). Thus, although the items of the euphoria scale load onto a single factor and have adequate internal consistency, it is unclear what construct these items measure. If anything, they seem to mostly capture positive aspects of mental health in youth, which contrasts findings with adults where this scale has been shown to be positively associated with interview-based indicators of mania and BD (Watson & O’Hara, 2017; Watson et al., 2012). The OCD scales also showed weaker correlations with functional impairment than the other IDAS-II scales, as well as the weakest ability to discriminate those with current mental health difficulties. This is in line with previous evaluations of the IDAS-II in adults and has been suggested to reflect subclinical manifestations of OCD-related behaviors and traits in the general population (Watson et al., 2012).

We examined the degree to which the IDAS-II could identify those who rated themselves as having ongoing mental health problems using a stringent two-step approach, with classification performance being based on data not used for coefficient estimation. To put results in context, we compared the classification performance of the IDAS-II with that of the RCADS. Overall, the IDAS-II showed excellent classification performance with a sensitivity of 82% and a PPV of 82%. Specificity and NPV were 78% for both indicators. The IDAS-II had better values than RCADS for all four indicators of classification performance, which is somewhat surprising since the different indicators often compete (i.e., higher sensitivity lower PPV). These results indicate that the IDAS-II may be a useful screening measure in clinical practice, but it should be noted that no formal statistical comparisons of classification performance were conducted because of low statistical power. Furthermore, the measure of current mental health difficulties was purely self-reported.

Several limitations of the current study deserve mentioning. First, as mentioned above, no diagnostic interviews were conducted and information about current mental health was purely self-reported. Future studies should include diagnostic interviews and other interview-based information on current mental health; information from caregivers would be valuable since youths and caregivers provide unique information in relation to mental health difficulties in children and adolescents (Aebi et al., 2017; De Los Reyes et al., 2015). Second, no repeated measurement was conducted, which precluded evaluation of temporal stability and sensitivity of the IDAS-II to change. More knowledge about the measure’s sensitivity to changes in mental health and everyday functioning following treatment is important for the measure to be used clinically. Third, the youth participants were anonymous and only limited personal information was collected. It is therefore hard to infer to what degree the current results can be generalized. For example, half of the youth sample reported that they experienced current mental health difficulties, suggesting that the survey disproportionately drew the attention of youth with such difficulties. Future research should be conducted with representative samples of children and adolescents, which would also allow for the calculation of norms. Finally, we had few participants in the lower age range. Nevertheless, we conducted separate analyses in those under 16 years of age and the results (i.e., model/data fit and internal consistency of each scale) were very similar when compared with older youths and the adult sample. Although this is promising, larger samples of younger youths are needed to provide more certainty about the suitability of IDAS-II in this age range.

In sum, the present study supports the use of the IDAS-II as a measure of internalizing symptom dimensions in children and adolescents. The psychometric properties of its 18 scales are consistent with the adult version; moreover, the scales correlate with mental health-related impairment in youth and can discriminate youths with current mental health difficulties from those without such difficulties. The measure shows promising clinical applicability, with excellent classification performance, and may be better at identifying youths with mental health problems than the currently recommended measure of youth internalizing symptoms. Future research should include more comprehensive clinical information, administer the IDAS-II repeatedly to examine its temporal stability and sensitivity to change in youths, and evaluate psychometric properties in younger youth samples.

## Supplemental Material

sj-docx-1-asm-10.1177_10731911231170841 – Supplemental material for A Psychometric Evaluation of the Expanded Version of the Inventory of Depression and Anxiety Symptoms (IDAS-II) in Children and Adolescents Supplemental material, sj-docx-1-asm-10.1177_10731911231170841 for A Psychometric Evaluation of the Expanded Version of the Inventory of Depression and Anxiety Symptoms (IDAS-II) in Children and Adolescents by Matti Cervin, Carla Martí Valls, Stefan Möller, Andreas Frick, Johannes Björkstrand and David Watson in Assessment

## Notes

1. Both versions of the IDAS also include a 20-item General Depression scale that includes items from several scales. It will not be examined in our study.

2. Since most previous studies have reported an overarching three-factor structure of the IDAS-II, we report the three-factor solution for youth: Factor 1: dysphoria, lassitude, insomnia, suicidality, appetite loss, appetite gain, ill temper, mania, panic, social anxiety, traumatic intrusions, traumatic avoidance; Factor 2: claustrophobia, checking, ordering, cleaning; Factor 3: well-being, euphoria.
