# Supplementary material for: A Psychometric Evaluation of the Expanded Version of the Inventory of Depression and Anxiety Symptoms (IDAS-II) in Children and Adolescents
Source: Assessment. 2023 May 12;31(3):588–601. doi: 10.1177/10731911231170841 (PMC10903129; doi:10.1177/10731911231170841)
Supplement: sj-docx-1-asm-10.1177_10731911231170841 – Supplemental material for A Psychometric Evaluation of the Expanded Version of the Inventory of Depression and Anxiety Symptoms (IDAS-II) in Children and Adolescents [file sj-docx-1-asm-10.1177_10731911231170841.docx]

**Table S1.** Standardized factor loadings for all IDAS-II items across samples.

| Scales | | Full Youth Sample | Youth  Survey 1 | Youth  Survey 2 | Adults |
| --- | --- | --- | --- | --- | --- |
| Dysphoria | |  |  |  |  |
|  | Low interest | 0.70 | 0.60 | 0.69 | 0.60 |
|  | Nervousness | 0.59 | 0.67 | 0.06 | 0.66 |
|  | Depression | 0.85 | 0.81 | 0.86 | 0.83 |
|  | Indecisiveness | 0.65 | 0.61 | 0.63 | 0.62 |
|  | Feelings of guilt | 0.77 | 0.80 | 0.78 | 0.80 |
|  | Feelings of inadequacy | 0.79 | 0.78 | 0.80 | 0.79 |
|  | Low motivation | 0.71 | 0.79 | 0.71 | 0.79 |
|  | Persistent worry | 0.81 | 0.81 | 0.81 | 0.81 |
|  | Slow speech | 0.65 | 0.65 | 0.65 | 0.65 |
|  | Low concentration | 0.74 | 0.80 | 0.72 | 0.80 |
| Lassitude | |  |  |  |  |
|  | Exhaustion | 0.83 | 0.82 | 0.85 | 0.82 |
|  | Oversleeping | 0.48 | 0.27 | 0.48 | 0.27 |
|  | Low energy | 0.81 | 0.86 | 0.78 | 0.86 |
|  | Problems waking up | 0.55 | 0.44 | 0.50 | 0.43 |
|  | Morning tiredness | 0.49 | 0.32 | 0.52 | 0.36 |
|  | Somnolence | 0.77 | 0.77 | 0.77 | 0.76 |
| Insomnia | |  |  |  |  |
|  | Sleep decrease | 0.74 | 0.58 | 0.74 | 0.58 |
|  | Difficulties falling asleep | 0.78 | 0.79 | 0.74 | 0.77 |
|  | Difficulties getting back to sleep | 0.68 | 0.88 | 0.71 | 0.89 |
|  | Waking up too early | 0.66 | 0.82 | 0.68 | 0.82 |
|  | Waking up during the night | 0.85 | 0.82 | 0.88 | 0.81 |
|  | Poor sleep quality | 0.90 | 0.88 | 0.91 | 0.87 |
| Suicidality | |  |  |  |  |
|  | Suicidal thoughts | 0.89 | 0.90 | 0.89 | 0.91 |
|  | Self-cutting | 0.95 | 0.94 | 0.94 | 0.97 |
|  | Feeling worthless | 0.95 | 0.90 | 0.97 | 0.89 |
|  | Self-harm | 0.97 | 0.93 | 0.96 | 0.94 |
|  | Thoughts of dying | 0.82 | 0.79 | 0.84 | 0.79 |
|  | Self-harming thoughts | 0.92 | 0.96 | 0.92 | 0.96 |
| Appetite Loss | |  |  |  |  |
|  | Poor appetite | 0.90 | 0.90 | 0.88 | 0.89 |
|  | Eating less than usual | 0.88 | 0.92 | 0.92 | 0.91 |
|  | Low desire to eat | 0.91 | 0.94 | 0.91 | 0.95 |
| Appetite Gain | |  |  |  |  |
|  | Food rumination | 0.79 | 0.84 | 0.80 | 0.79 |
|  | Eating without appetite | 0.64 | 0.58 | 0.66 | 0.58 |
|  | Eating more than usual | 0.88 | 0.89 | 0.92 | 0.94 |
| Well-being | |  |  |  |  |
|  | Optimism | 0.83 | 0.86 | 0.84 | 0.86 |
|  | Proud of oneself | 0.81 | 0.79 | 0.82 | 0.78 |
|  | Self-recognition | 0.79 | 0.72 | 0.81 | 0.69 |
|  | Excitement | 0.85 | 0.85 | 0.86 | 0.85 |
|  | Hopefulness | 0.81 | 0.89 | 0.84 | 0.90 |
|  | Have things to look forward to | 0.87 | 0.88 | 0.87 | 0.87 |
|  | High interest | 0.73 | 0.81 | 0.76 | 0.80 |
|  | High energy | 0.70 | 0.75 | 0.69 | 0.73 |
| Ill Temper | |  |  |  |  |
|  | Feeling furious | 0.82 | 0.72 | 0.84 | 0.71 |
|  | Breaking things | 0.78 | 0.90 | 0.83 | 0.93 |
|  | Feeling enraged | 0.82 | 0.86 | 0.82 | 0.85 |
|  | Shouting at people | 0.80 | 0.74 | 0.83 | 0.73 |
|  | Irritability | 0.90 | 0.84 | 0.89 | 0.84 |
| Mania | |  |  |  |  |
|  | High distractibility | 0.47 | 0.39 | 0.57 | 0.39 |
|  | Unintelligible speech | 0.65 | 0.70 | 0.69 | 0.72 |
|  | Racing thoughts | 0.92 | 0.93 | 0.90 | 0.94 |
|  | Quick change of thoughts | 0.71 | 0.74 | 0.78 | 0.74 |
|  | Not keeping up with own thoughts | 0.92 | 0.89 | 0.94 | 0.88 |
| Euphoria | |  |  |  |  |
|  | Elated with no reason | 0.77 | 0.75 | 0.74 | 0.74 |
|  | Overexcitement | 0.79 | 0.86 | 0.74 | 0.85 |
|  | Hyperactivity | 0.65 | 0.84 | 0.71 | 0.83 |
|  | Inflated self-esteem | 0.56 | 0.54 | 0.56 | 0.51 |
|  | Higher energy than usual | 0.71 | 0.83 | 0.70 | 0.82 |
| Panic | |  |  |  |  |
|  | Chest pain | 0.82 | 0.82 | 0.81 | 0.81 |
|  | Dizziness | 0.79 | 0.75 | 0.76 | 0.75 |
|  | Shaking | 0.84 | 0.86 | 0.85 | 0.86 |
|  | Fainting | 0.81 | 0.81 | 0.79 | 0.80 |
|  | Increased heartbeat | 0.77 | 0.85 | 0.76 | 0.85 |
|  | Dry mouth | 0.73 | 0.66 | 0.72 | 0.64 |
|  | Breathlessness | 0.86 | 0.86 | 0.86 | 0.86 |
|  | Choking sensation | 0.88 | 0.87 | 0.89 | 0.87 |
| Social Anxiety | |  |  |  |  |
|  | Self-consciousness | 0.63 | 0.57 | 0.58 | 0.58 |
|  | Embarrassment | 0.76 | 0.78 | 0.74 | 0.77 |
|  | Claustrophobia | 0.88 | 0.91 | 0.87 | 0.90 |
|  | Difficulties making eye-contact | 0.74 | 0.77 | 0.77 | 0.78 |
|  | Fear of strangers | 0.69 | 0.78 | 0.69 | 0.77 |
|  | Fear of public speaking | 0.67 | 0.77 | 0.69 | 0.75 |
| Claustrophobia | |  |  |  |  |
|  | Avoid small spaces | 0.89 | 0.91 | 0.87 | 0.92 |
|  | Fear of being trapped | 0.94 | 1.03 | 0.91 | 1.02 |
|  | Anxious in small spaces | 0.93 | 0.93 | 0.96 | 0.93 |
|  | Fear of tunnels | 0.65 | 0.74 | 0.66 | 0.77 |
|  | Avoid enclosed spaces | 0.91 | 0.88 | 0.91 | 0.88 |
| Traumatic Intrusions | |  |  |  |  |
|  | Disturbing thoughts of something bad that has happened to me | 0.85 | 0.92 | 0.86 | 0.93 |
|  | Nightmares reminding me | 0.74 | 0.71 | 0.76 | 0.71 |
|  | Memories of something scary | 0.88 | 0.92 | 0.88 | 0.92 |
|  | Upset thinking about it | 0.89 | 0.90 | 0.90 | 0.89 |
| Traumatic Avoidance | |  |  |  |  |
|  | Tried to not think of bad things from the past | 0.85 | 0.86 | 0.86 | 0.88 |
|  | Avoid situations that bring up bad memories | 0.90 | 0.87 | 0.89 | 0.86 |
|  | Ignore upsetting memories | 0.90 | 0.89 | 0.90 | 0.90 |
|  | Avoid talking about bad experiences from past | 0.68 | 0.66 | 0.69 | 0.68 |
| Checking | |  |  |  |  |
|  | Checking repeatedly | 0.80 | 0.83 | 0.84 | 0.84 |
|  | Checking even when not necessary | 0.88 | 0.82 | 0.91 | 0.81 |
|  | Urge to make sure I have done something | 0.82 | 0.84 | 0.85 | 0.84 |
| Ordering | |  |  |  |  |
|  | Rearrange things | 0.60 | 0.63 | 0.64 | 0.62 |
|  | Rearrange until ‘just right’ | 0.80 | 0.84 | 0.81 | 0.84 |
|  | Fixed order in everyday tasks | 0.69 | 0.60 | 0.74 | 0.61 |
|  | Compelled to follow rituals | 0.85 | 0.86 | 0.83 | 0.85 |
|  | Time consuming rituals or habits | 0.83 | 0.71 | 0.80 | 0.73 |
| Cleaning | |  |  |  |  |
|  | Excessive handwashing | 0.79 | 1.01 | 0.82 | 1.01 |
|  | Worry about germs | 0.80 | 0.81 | 0.79 | 0.82 |
|  | Avoid touching dirty things | 0.93 | 0.90 | 0.95 | 0.89 |
|  | Difficulties touching dirty things | 0.91 | 0.88 | 0.92 | 0.87 |
|  | Fear of contamination | 0.82 | 0.76 | 0.82 | 0.77 |
|  | Avoid public restrooms | 0.65 | 0.73 | 0.69 | 0.75 |
|  | Use objects to avoid touching things | 0.76 | 0.67 | 0.78 | 0.69 |

**Table S2.** Internal consistency of the items of each IDAS-II factor in the youth and adult samples.

|  | Full Youth  Sample | | Youth  Survey 1 | | Youth  Survey 2 | | Adult Sample | |
| --- | --- | --- | --- | --- | --- | --- | --- | --- |
|  | Alpha | Omega | Alpha | Omega | Alpha | Omega | Alpha | Omega |
| Dysphoria | .91 | .90 | .91 | .90 | .91 | .90 | .92 | .90 |
| Lassitude | .82 | .79 | .82 | .78 | .82 | .80 | .78 | .72 |
| Insomnia | .88 | .87 | .88 | .88 | .87 | .86 | .87 | .89 |
| Suicidality | .95 | .95 | .95 | .95 | .95 | .94 | .95 | .93 |
| Appetite Loss | .92 | .90 | .93 | .90 | .91 | .89 | .94 | .92 |
| Appetite Gain | .76 | .77 | .76 | .80 | .74 | .72 | .81 | .79 |
| Well-being | .93 | .92 | .94 | .92 | .92 | .91 | .93 | .92 |
| Ill temper | .91 | .89 | .92 | .90 | .88 | .86 | .90 | .88 |
| Mania | .86 | .84 | .87 | .86 | .81 | .78 | .86 | .84 |
| Euphoria | .80 | .76 | .80 | .76 | .81 | .77 | .86 | .78 |
| Panic | .93 | .92 | .93 | .91 | .94 | .92 | .93 | .91 |
| Social Anxiety | .86 | .85 | .86 | .84 | .87 | .85 | .89 | .86 |
| Claustrophobia | .90 | .92 | .90 | .92 | .90 | .93 | .94 | .94 |
| Traumatic intrusions | .89 | .87 | .89 | .88 | .87 | .86 | .91 | .89 |
| Traumatic avoidance | .88 | .87 | .89 | .87 | .88 | .87 | .89 | .87 |
| Checking | .87 | .84 | .90 | .87 | .81 | .77 | .86 | .82 |
| Ordering | .87 | .82 | .87 | .83 | .86 | .81 | .83 | .78 |
| Cleaning | .92 | .90 | .92 | .91 | .91 | .89 | .92 | .90 |

**Table S3.** Internal consistency of the items of each IDAS-II factor in the younger and older youth samples.

|  | Younger Youth  (< 16 years) | | Older Youth  (16-19 years) | |
| --- | --- | --- | --- | --- |
|  | Alpha | Omega | Alpha | Omega |
| Dysphoria | .90 | .89 | .91 | .90 |
| Lassitude | .82 | .80 | .83 | .79 |
| Insomnia | .87 | .85 | .88 | .88 |
| Suicidality | .95 | .95 | .95 | .95 |
| Appetite Loss | .94 | .91 | .92 | .89 |
| Appetite Gain | .76 | .76 | .75 | .76 |
| Well-being | .92 | .91 | .93 | .92 |
| Ill temper | .90 | .88 | .91 | .89 |
| Mania | .88 | .87 | .85 | .83 |
| Euphoria | .76 | .73 | .81 | .76 |
| Panic | .94 | .92 | .93 | .92 |
| Social Anxiety | .89 | .88 | .85 | .84 |
| Claustrophobia | .91 | .93 | .90 | .91 |
| Traumatic intrusions | .92 | .89 | .88 | .87 |
| Traumatic avoidance | .92 | .91 | .88 | .86 |
| Checking | .89 | .85 | .86 | .83 |
| Ordering | .88 | .85 | .86 | .82 |
| Cleaning | .90 | .89 | .92 | .90 |

**Table S4.** 2 by 2 tables for classification performance of IDAS-II and RCADS.

| IDAS-II | Current mental health problems | No current mental health problems |
| --- | --- | --- |
| Classified as having current mental health problems | 53 | 12 |
| Classified as not having current mental health problems | 12 | 43 |
| RCADS | Current mental health problems | No current mental health problems |
| Classified as having current mental health problems | 52 | 18 |
| Classified as not having current mental health problems | 14 | 38 |
